# Partial Segmental Thrombosis of the Corpus Cavernosum Following Valsalva Maneuver: A Case Report

**DOI:** 10.5811/cpcem.65781

**Published:** 2026-10-13

**Authors:** Sitha Konopack, Jessica Diaz

**Affiliations:** University of Florida College of Medicine, Department of Emergency Medicine, Gainesville, Florida

**Keywords:** emergency department, pelvic pain, corpus cavernosum thrombosis, groin pain, case report

## Abstract

**Introduction:**

Partial segmental thrombosis of the corpus cavernosum is a rare vascular condition that may present with nonspecific groin or pelvic pain and mimic more common surgical or urologic emergencies.

**Case Report:**

A 33-year-old man presented to the emergency department with acute left-sided groin and pelvic pain after straining during defecation. Initial history and evaluation raised concern for incarcerated inguinal hernia, epididymo-orchitis, renal colic, lymphadenopathy, and soft tissue infection. Computed tomography demonstrated asymmetric enlargement of the left corpus cavernosum, while penile ultrasound showed diminished cavernosal flow. Magnetic resonance imaging confirmed segmental thrombosis of the left corpus cavernosum. The patient was treated conservatively with rivaroxaban and analgesia, resulting in symptomatic improvement.

**Conclusion:**

Partial segmental thrombosis of the corpus cavernosum should be considered in patients presenting with unexplained groin or perineal pain when standard abdominal, penile, testicular, and hernia evaluations are unrevealing. Magnetic resonance imaging may be necessary for definitive diagnosis, and conservative management is frequently successful.

## INTRODUCTION

Groin and lower pelvic pain are common emergency department (ED) presentations with a broad differential diagnosis, including inguinal hernia, urologic infection, renal colic, testicular torsion, musculoskeletal injury, paraphimosis, priapism, and soft tissue infection. Vascular causes involving penile structures are often overlooked during initial evaluation.

Partial segmental thrombosis of the corpus cavernosum (PSTCC) is an uncommon vascular disorder characterized by localized thrombosis within the erectile tissue of the penis and has rarely been described in the literature.^1^–^5^ Unlike ischemic priapism, PSTCC often presents without sustained erection, contributing to delayed diagnosis or misdiagnosis.^1^–^3^

We describe a case of PSTCC presenting as acute groin and pelvic pain following a Valsalva maneuver during defecation, highlighting the diagnostic challenges and imaging findings relevant to emergency medicine practice.

## CASE REPORT

A 33-year-old male with a history of prior left inguinal hernia repair presented to the ED with 3 days of progressive left groin and pelvic pain that began immediately after straining during defecation. He reported difficulty passing stool and transient nonbloody emesis but denied fever, urinary symptoms, hematuria, penile discharge, trauma, erectile dysfunction, or sustained erection. He denied a personal or family history of thromboembolic disease.

On presentation, his vital signs were as follows: blood pressure, 116/82 mm Hg; heart rate, 67 beats per minute; respiratory rate, 15 breaths per minute; temperature, 36.7 °C; and SpO_2_, 99% on room air. Physical examination demonstrated a well-appearing male in no acute distress. The abdomen was soft, nontender, and nondistended without guarding or rebound tenderness. Examination of the external genitalia revealed a normal, flaccid penis without swelling, tenderness, erythema, discoloration, or priapism. A small palpable nodule with minimal tenderness was identified in the left perineum. Bilateral testes were descended and equal in size, with mild tenderness to palpation of the left testicle. There was no scrotal swelling, crepitus, erythema, skin discoloration, or other evidence of soft tissue infection involving the perineum. The initial emergency department differential diagnosis included incarcerated inguinal hernia, epididymo-orchitis, renal colic, lymphadenopathy, and soft tissue infection.

Computed tomography (CT) of the abdomen and pelvis demonstrated no intra-abdominal pathology or recurrent hernia but did reveal asymmetric enlargement of the left corpus cavernosum (Image 1).

Subsequent penile ultrasound demonstrated a heterogeneous avascular lesion within the left corpus cavernosum, raising concern for cavernosal vascular pathology (Image 2).

Given the patient’s persistent pain and indeterminate imaging findings, urology consultation was obtained, and further evaluation with magnetic resonance imaging (MRI) and cavernosal blood gas analysis was recommended. The cavernosal blood gas demonstrated a markedly hypoxic sample with low oxygen tension and oxygen saturation, consistent with localized cavernosal ischemia and supportive of the diagnosis of partial segmental thrombosis of the corpus cavernosum (Table).

Magnetic resonance imaging of the pelvis subsequently confirmed a 7-cm segment of thrombosis involving the left corpus cavernosum with absent postcontrast enhancement consistent with thrombus (Image 3).

Cavernosal blood gas analysis obtained in the ED demonstrated hypoxic venous blood, supporting localized ischemia. The final diagnosis was partial segmental thrombosis of the corpus cavernosum. The patient was started on an intravenous heparin infusion and admitted to the family medicine service with ongoing urology and hematology consultation.

*CPC-EM Capsule*

What do we already know about this clinical entity?

*Partial segmental thrombosis of the corpus cavernosum is a rare penile vascular disorder that often presents without classic priapism.*

What makes this presentation of disease reportable?

*We report a case of isolated groin and pelvic pain following a Valsalva maneuver, mimicking more common surgical and urologic conditions.*

What is the major learning point?

*Partial segmental thrombosis of the corpus cavernosum should be considered in patients with unexplained groin or perineal pain and unrevealing initial evaluation.*

How might this improve emergency medicine practice?

*Recognition of this uncommon diagnosis may facilitate appropriate imaging, specialty consultation, and timely conservative management.*

The patient was managed collaboratively with urology and hematology consultation. Initial treatment consisted of intravenous heparin infusion for anticoagulation along with ibuprofen for analgesia. Following clinical stabilization, the patient was transitioned to rivaroxaban 20 mg daily with a planned treatment duration of 3 months. No surgical intervention was required. The patient reported symptomatic improvement prior to discharge and was discharged with outpatient urology and hematology follow-up. No adverse events occurred during hospitalization. Outpatient evaluation for hypercoagulability revealed no provoking factors, and hypercoagulable testing was negative. The patient completed a three-month course of rivaroxaban with continued symptomatic improvement but was subsequently lost to follow-up.

## DISCUSSION

From an emergency medicine perspective, PSTCC is an uncommon diagnosis that may not be initially considered in patients presenting with isolated groin or perineal pain. Patients frequently lack the prolonged erection or obvious penile findings associated with ischemic priapism, and the diagnosis may be overlooked during the initial ED evaluation. Initial assessment appropriately prioritizes more common and time-sensitive conditions, including incarcerated inguinal hernia, urologic infection, renal colic, testicular torsion, musculoskeletal injury, paraphimosis, priapism, and soft tissue infection. The palpable inguinal abnormality in this case may have represented referred tenderness or extension along proximal corporal structures rather than discrete superficial pathology, although the precise correlation between the physical examination finding and imaging abnormality remained uncertain.

Partial segmental thrombosis of the corpus cavernosum is a rare vascular condition that characteristically presents with localized penile, groin, or perineal pain without the prolonged erection typical of an ischemic priapism.^1^–^3^ The condition is thought to result from venous outflow obstruction within a septated segment of the corpus cavernosum, leading to localized thrombosis.^2^ Valsalva maneuvers, including straining during defecation, are proposed precipitating factors because transient increases in intracavernosal venous pressure may promote venous stasis and thrombosis.^1^–^3^ Superficial dorsal penile vein thrombosis, also known as Mondor disease, may present with similar penile or groin pain but typically involves the superficial dorsal venous system rather than cavernosal tissue.^2^

Unlike ischemic priapism, PSTCC often involves only a segment of the corpus cavernosum due to the presence of a fibrous septum, thereby preventing complete corporal involvement and sustained erection. Diagnosis is primarily imaging-based. Ultrasound may demonstrate an avascular heterogeneous lesion, while MRI provides superior characterization of thrombus extent and exclusion of alternative penile pathology.^2^ Although CT demonstrated asymmetric cavernosal enlargement, the finding was nonspecific, whereas MRI provided definitive characterization of the thrombus and exclusion of alternative pathology. Cavernosal blood gas analysis may demonstrate hypoxia consistent with localized ischemia. Management is typically conservative and consists of anticoagulation and nonsteroidal anti-inflammatory medications, with most patients experiencing favorable outcomes.^1^–^4^

For emergency physicians, this case highlights the importance of including penile vascular pathology in the differential diagnosis of patients presenting with unexplained groin or perineal pain. Unrevealing abdominal imaging should not exclude consideration of deep pelvic or penile vascular pathology, particularly when symptoms persist despite an otherwise reassuring evaluation. Correlation of focal inguinal or perineal examination findings with advanced imaging may facilitate earlier diagnosis and appropriate conservative management.

## CONCLUSION

Partial segmental thrombosis of the corpus cavernosum is an uncommon but important vascular cause of groin and perineal pain that may mimic more common surgical or urologic conditions. Emergency physicians should consider PSTCC when standard abdominal, testicular, and hernia evaluations are unrevealing. Magnetic resonance imaging may be required for definitive diagnosis, and conservative management with anticoagulation is often successful.

## Figures and Tables

**Image 1:**
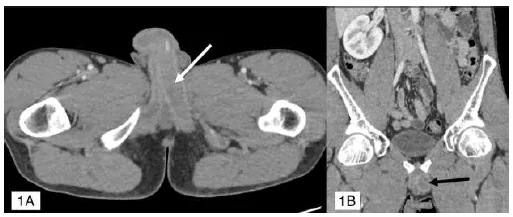
Computed tomography of the abdomen and pelvis with intravenous contrast. (**A**) Axial view demonstrates asymmetric enlargement of the left corpus cavernosum, highlighting changes in cross-sectional diameter of the cavernous body (white arrow). (**B**) Coronal view demonstrates longitudinal asymmetry of the left corpus cavernosum, demonstrating the cranial-to-caudal extent of the enlargement (black arrow).

**Image 2:**
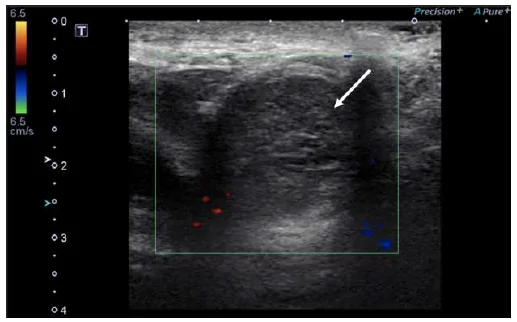
Penile ultrasound. Grayscale ultrasound demonstrates heterogeneous avascular lesion within the left corpus cavernosum (arrow).

**Image 3:**
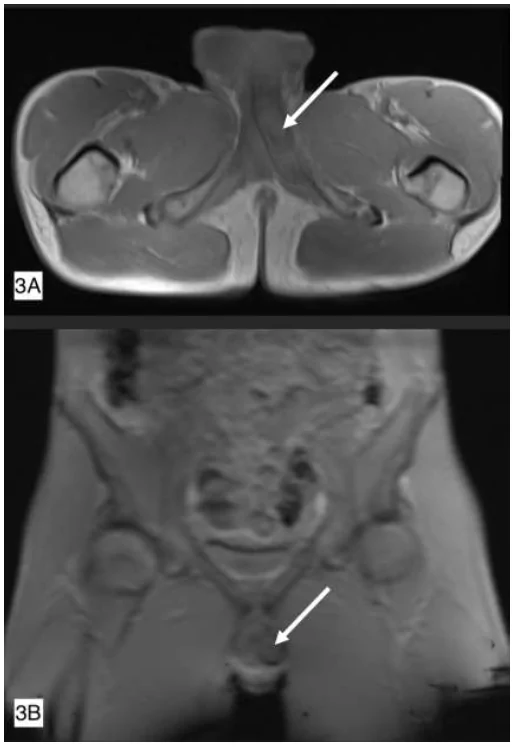
Magnetic resonance imaging of the pelvis. **(A)** Axial view demonstrates asymmetry between the left and right corpora cavernosa, with localized, segmental expansion of the left corpus cavernosum. The white arrow highlights widening and an absence of typical blood flow or internal contrast enhancement within the affected tissue. **(B)** Coronal view demonstrates asymmetric, segmental enlargement of the left corpus cavernosum (white arrow), with a distinct T2-hypointense signal in the proximal portion and mass effect on the adjacent right (contralateral) corpus cavernosum.

**Table:** Cavernosal blood gas analysis.

Parameter	Result
pH	7.46
pCO_2_	37 mmHg
pO_2_	35 mmHg
HCO_3_^−^	26 mmol/L
Base Excess	+2 mmol/L
SpO_2_	70%
Total CO_2_	27 mmol/L

*CO*_2_, carbon dioxide*; HCO*_3_, bicarbonate; *mmHg*, millimeters of mercury; *mmol/L*, millimoles per liter; *pCO*_2_, partial pressure of carbon dioxide; *pO*_2_, partial pressure of oxygen; SpO_2_, oxygen saturation.
